# Molecular interaction of fibrinogen with zeolite nanoparticles

**DOI:** 10.1038/s41598-018-37621-4

**Published:** 2019-02-07

**Authors:** Hossein Derakhshankhah, Atiyeh Hosseini, Fereshteh Taghavi, Samira Jafari, Alireza Lotfabadi, Mohammad Reza Ejtehadi, Sahba Shahbazi, Ali Fattahi, Atiyeh Ghasemi, Ebrahim Barzegari, Mina Evini, Ali Akbar Saboury, Seyed Mehdi Kamali Shahri, Behnaz Ghaemi, Eng-Poh Ng, Hussein Awala, Fatemeh Omrani, Iraj Nabipour, Mohammad Raoufi, Rassoul Dinarvand, Koorosh shahpasand, Svetlana Mintova, Mohammad Javad Hajipour, Morteza Mahmoudi

**Affiliations:** 1 Nano Drug Delivery Research Center, Kermanshah University of Medical Sciences, Kermanshah, Iran; 2 Pharmacutical Sciences Research Center, Kermanshah University of Medical Sciences, Kermanshah, Iran; 3 Institute for Nanoscience and Nanotechnology and Center of Excellence in Complex Systems and Condensed Matter (CSCM), Sharif University of Technology, Tehran, 1458889694 Iran; 4 Institute of Biochemistry and Biophysics, University of Tehran, Tehran, Iran; 5 Department of Physics, Sharif University of Technology, P. O. Box 11155-9161, Tehran, Iran; 6 Center of Excellence in Complex Systems and Condensed Matter (CSCM), Sharif University of Technology, Tehran, 1458889694 Iran; 7 School of Biology College of Science, University of Tehran, Tehran, Iran; 8 Department of Chemical Engineering, Pennsylvania State University, University Park, PA 16802 United States; 9 Department of Medical Nanotechnology, School of Advanced Technologies in Medicine (SATiM), Tehran University of Medical Sciences, Tehran, 1417755469 Iran; 10 School of Chemical Sciences, Universiti Sains Malaysia, Gelugor, 11800 USM Malaysia; 11 Laboratory of Catalysis and Spectroscopy, ENSICAEN, University of Caen, CNRS, 6 Boulevard du Marechal Juin, 14050 Caen, France; 12 Persian Gulf Marine Biotechnology Research Center, the Persian Gulf Biomedical Sciences Research Institute, Bushehr University of Medical Sciences, Bushehr, 75147 Iran; 13 Nanotechnology Research Center, Faculty of Pharmacy, Department of Pharmaceutical Nanotechnology, Faculty of Pharmacy, Tehran University of Medical Sciences, Tehran, 13169-43551 Iran; 14 Department of Brain and Cognitive Sciences, Cell Science Research Center, Royan Institute for Stem Cell Biology and Technology, ACECR, Tehran, Iran; 15 Non-Communicable Diseases Research Center, Endocrinology and Metabolism Population Sciences Institute, Tehran University of Medical Sciences, Tehran, 13169-43551 Iran; 16 Department of Anesthesiology, Brigham and Women’s Hospital, Harvard Medical School, Boston, Massachusetts, 02115 United States

## Abstract

Fibrinogen is one of the key proteins that participate in the protein corona composition of many types of nanoparticles (NPs), and its conformational changes are crucial for activation of immune systems. Recently, we demonstrated that the fibrinogen highly contributed in the protein corona composition at the surface of zeolite nanoparticles. Therefore, understanding the interaction of fibrinogen with zeolite nanoparticles in more details could shed light of their safe applications in medicine. Thus, we probed the molecular interactions between fibrinogen and zeolite nanoparticles using both experimental and simulation approaches. The results indicated that fibrinogen has a strong and thermodynamically favorable interaction with zeolite nanoparticles in a non-cooperative manner. Additionally, fibrinogen experienced a substantial conformational change in the presence of zeolite nanoparticles through a concentration-dependent manner. Simulation results showed that both E- and D-domain of fibrinogen are bound to the EMT zeolite NPs *via* strong electrostatic interactions, and undergo structural changes leading to exposing normally buried sequences. D-domain has more contribution in this interaction and the C-terminus of γ chain (γ^377–394^), located in D-domain, showed the highest level of exposure compared to other sequences/residues.

## Introduction

Nanoparticles (NPs) have potential application for disease diagnosis and therapy^1–5^. It is well-recognized that the NP surfaces are instantaneously covered by a layer of biomolecules (so-called protein corona), when they come into contact with physiological fluids such as blood plasma^6–9^. The competitive adsorption of proteins on the NP surface is dependent on the physicochemical properties of NPs, incubation condition (time and temperature), and plasma protein alterations (protein concentration/structure) mediated by diseases^9–17^. In fact, the formation of protein corona changes the surface properties of NPs and it provides a biological mask, which is being “seen” by the biological systems such as cells^7,18–21^. The type, concentration and configuration of the participated proteins in the corona layer can give useful information for predicting the biological fate of NPs including their pharmacokinetics and biodistribution^10,14,22–25^.

During last few years, many studies were devoted on achieving in-depth information on the structural integrity of proteins after participating in the corona structure. As expected, the preliminary results revealed that the degree of structural rearrangement in protein is dependent on the protein type and physicochemical properties of NPs^14,26,27^. As an example of the role of protein type, fibrinogen demonstrated higher structural changes compared to albumin when interacting with graphene oxide^14^ and silica NPs^27^. Regarding the NPs’ properties, hydrophobic surfaces induce more structural changes to both albumin and fibrinogen compared to hydrophilic surfaces^27^.

Moreover, the structural variations of proteins can change their physiological functions. For example, we demonstrated that transferrin experiences irreversible structural changes and loose the main functionality (i.e., transport of iron among cells) after adsorption to the surface of iron oxide NPs^28^. Along with the functional changes, the structural changes in some proteins may activate inflammatory responses. For instance, structural changes in fibrinogen (i.e., exposure of its C-terminus of γ chain (γ^377–395^)), after interaction with poly(acrylic acid)-coated gold NPs, can provoke the inflammation response and downstream unwanted cascade pathways^24^.

Recently, we demonstrated that fibrinogen has the most contribution, among other plasma proteins, in the corona structure of the zeolite NPs^29,30^. Therefore, it is required to evaluate the structural integrity of zeolite-bound fibrinogen. To date, the molecular interactions between zeolite NPs and fibrinogen are not completely understood. The aim of this work is to understand the interactions of zeolite nanoparticles with fibrinogen using a wide range of theoretical and experimental approaches for probing the binding kinetics, and thermodynamic parameters and structural changes.

## Results and Discussion

### Characterization of EMT zeolite nanoparticles

**Figure 1:**
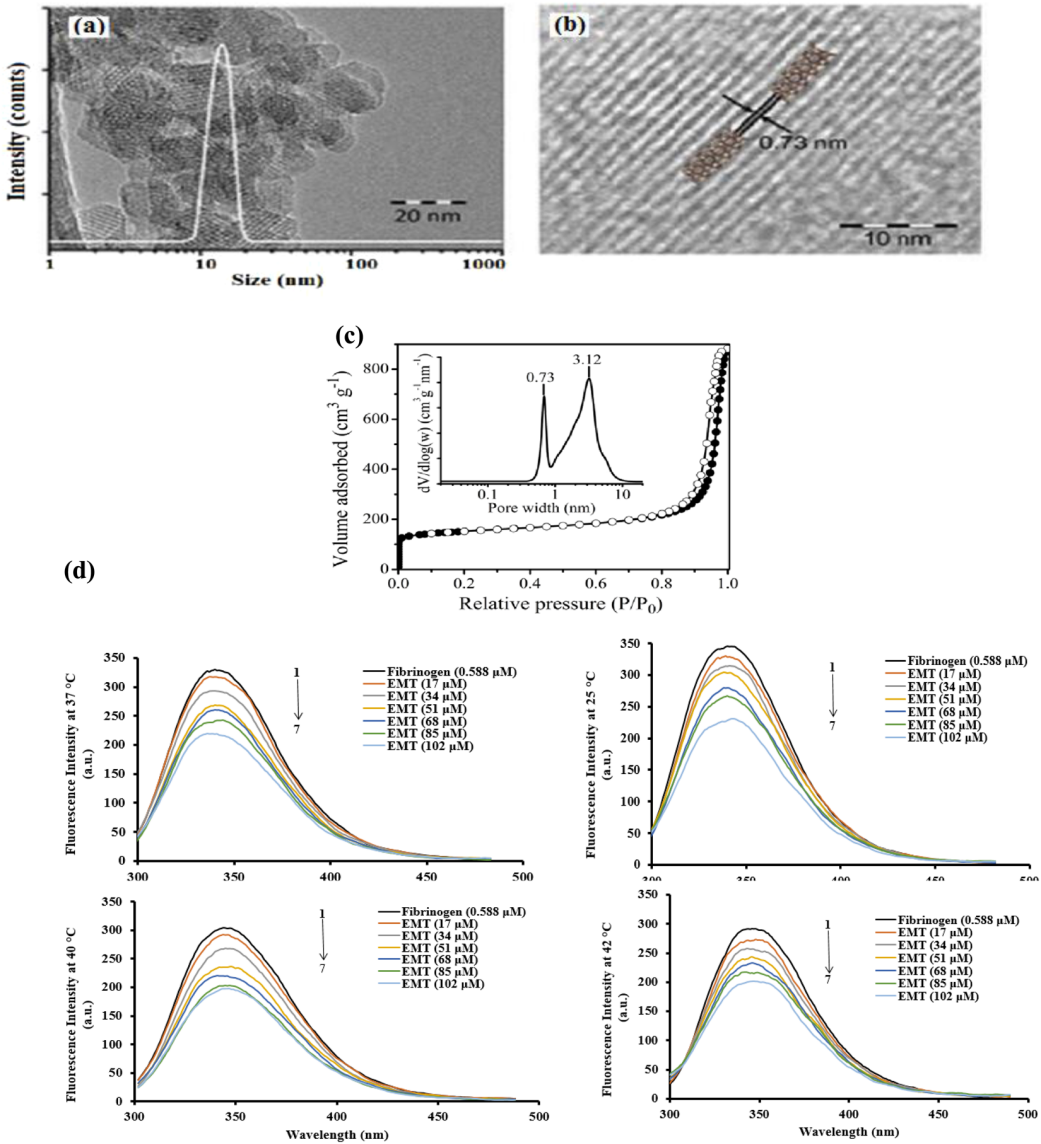
(**a**) TEM image and DLS curve and (**b**) crystalline fringes representing the porosity of EMT zeolite NPs in the TEM. (**c**) Nitrogen adsorption (close symbols) and desorption (open symbols) isotherm of EMT zeolite NPs. *Inset:* Pore size distribution derived from nitrogen sorption analysis using DFT model. (**d**) Fluorescence intensity of fibrinogen in the presence of different concentrations of EMT zeolite NPs at 25, 37, 40 and 42 °C.

Highly crystalline hexagonal EMT type zeolite NPs were synthesized and stabilized in water suspensions^31,32^. The particle size and the morphology of NPs were determined by dynamic light scattering (DLS) and transmission electron microscopy (TEM) techniques. The EMT nanocrystals indicated unimodal particle size distribution (8–20 nm) and hexagonal morphology (Fig. 1a,b). The porosity of the EMT zeolite was also measured. The EMT nanocrystals showed a combination of Types I and IV adsorption isotherm curves, indicating the presence of both micro- and textural meso- porosities (Fig. 1c)^33^ The size of the micropores determined using the DFT was 0.73 nm, which is in agreement with the size of the hypocage (0.75 nm × 0.65 nm in diameter) and hypercage (0.73 nm × 0.73 nm in diameter) of the EMT-type zeolite. In addition, the size of the mesopores was 3.1 nm, which is due to the close packing of the nanoparticles resulting in textural (inter-particles) porosity. The EMT zeolite nanoparticles have a BET surface area of 720 m^2^ g^−1^, external surface area of 260 m^2^ g^−1^, and total pore volume of 1.32 cm^3^ g^−1^. The chemical composition of the EMT zeolite was analysed using XRF spectroscopy. The unit cell composition was Na_88_ (AlO_2_)_88_(SiO_2_)_104,_ with the Si/Al ratio equal to 1.17, suggesting that the zeolite surface was highly negatively charged. Hence, the high Na content was detected to counter balance the negative charge originated from the (Al–O–Si)^−^ groups. The surface charge and the charge density of EMT NPs were also calculated (see Methods section). The result showed that the EMT zeolite NPs contained a surface charge of −4356 mC g^−1^ due to the high surface area and high alumina content in the framework. As a result, the high surface charge density (−6.05 mC m^−2^) was calculated (Table 1). The high surface charge and high charge density of EMT NPs are thus in line with their hydrophilic and polar nature.

**Table 1:** Physicochemical characteristics of EMT zeolite NPs.

Si/Al ratio	1.17
Unit cell composition	Na_88_(AlO_2_)_88_(SiO_2_)_104_
S_BET_ (m^2^ g^−1^)^a^	720
S_Ext_ (m^2^ g^−1^)^a^	260
V_micro_ (cm^3^ g^−1^)^a^	0.24
V_meso_ (cm^3^ g^−1^)^a^	1.08
V_Total_ (cm^3^ g^−1^)^a^	1.32
d_micro_ (nm)^a^	0.73
d_meso_ (nm)^a^	3.12
Mean particle size (nm)^b^	14.0
Charge density (mC m^−2^)^c^	−6.05
Surface charge (mC g^−1^)^d^	−4356

^a^SBET: BET specific surface area; Sext: external surface area; Vmicro: micropore volume; Vmeso: mesopore volume; Vtotal: total pore volume; dmicro: micropore diameter; dmeso: mesopore diameter.

^b^Determined by DLS and TEM.

^c^Determined at 0.1 wt% concentration.

^d^Determined at 0.1 wt% concentration.

### Interaction of fibrinogen with EMT zeolite nanoparticles

Fluorescence spectroscopy is widely used to study the interaction of NPs and proteins^34^. It is known that the intrinsic tryptophan (TRP) fluorescence has significant changes through protein unfolding, leading to the exposure of internal TRP that is typically hidden in the folded state^35^. We probed fibrinogen-folding variations during the interaction with zeolite NPs using the leverage of TRP *via* monitoring the protein folding changes. The fluorescence spectra of fibrinogen were measured in the presence of various concentrations of EMT NPs at different temperatures (25, 37, 40 and 42 °C) (Fig. 1d). The maximum fluorescence peak (λ_max_) of fibrinogens incubated with EMT NPs was observed at 342 nm, and the fluorescence intensity of fibrinogen gradually decreased as the zeolite NPs concentration increased. These results indicate that the EMT NPs, which act as a quencher, have strong interactions with the fibrinogen. In this case, one may speculate that zeolite-bounded fibrinogen may experience structural rearrangement leading to the change in microenvironment of internal TRP. Molecular interaction between protein and zeolite NPs assists to perceive the origins in addition to have safe zeolites with more predictable biological efficacy. Therefore, several parameters involved in the fibrinogen-zeolite interaction are probed as described in the following sections.

### Quenching behavior of EMT zeolite nanoparticles

**Figure 2:**
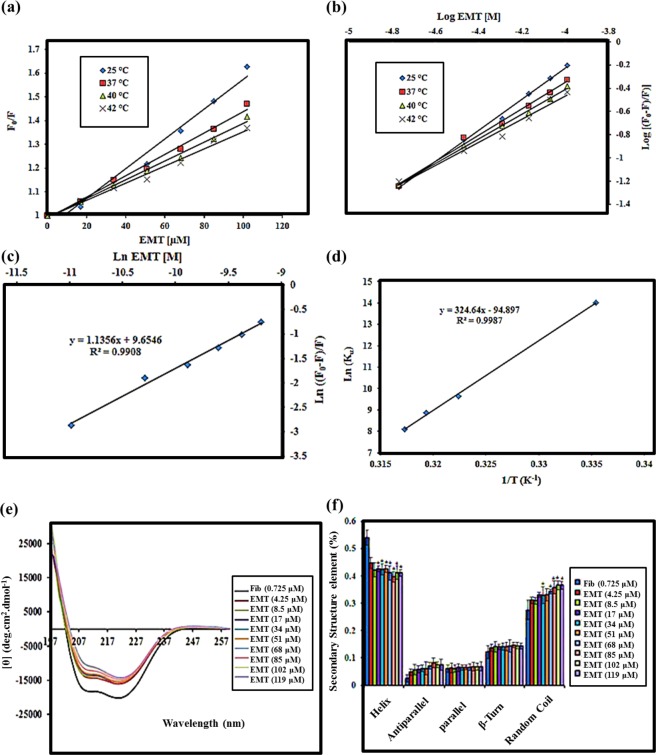
(**a**) The Stern - Volmer plots of fibrinogen quenching caused by different concentrations of EMT zeolite NPs at different temperatures. (**b**) The double-log plots Log ((F_0_ − F)/F) vs. Log EMT for fibrinogen–EMT zeolite NPs interactions at different temperatures. (**c**) The Hill plots Ln (F_0_ − F)/F)) vs. Ln EMT for fibrinogen–EMT zeolite NPs interactions at physiological temperature (37 °C). (**d**) The van’t Hoff plots of fibrinogen–EMT zeolite NPs interactions. (**e**) CD spectra of fibrinogen molecules alone and in the presence of different concentrations of EMT zeolite NPs. (**f**) Secondary structural changes of fibrinogen in the presence of increasing concentrations of the EMT zeolite NPs. Results are mean ± standard error of mean (*n* = 20). Star represents significant change compared to control (Fibrinogen alone) at p < 0.05.

It is well-recognized that NPs quench the proteins in a dynamic or static manner^36^. Depending on the incubation temperature, the fluorophore–quencher complex is formed before (static) and/or after (dynamic) fluorophore excitation^37^. The quenching behavior of NPs is strongly dependent on the temperature at which proteins are incubated with NPs. The molecular mechanism of quenching is proposed using the Stern-Volmer equation (SI, Eq. S1). The Stern-Volmer plots and the constants of fibrinogen quenching induced by different concentrations of EMT NPs at various temperatures are shown in Fig. 2a and Table 2, respectively. As can be seen, a decrease in both the Stern-Volmer quenching constants and the slope of the Stern–Volmer plot is observed at higher temperatures. This result indicates that fibrinogen is mainly quenched in a static manner. Previously, we showed that slight change in incubation temperature affects the protein decoration on the surface of NPs and consequent biological responses^38^. Depending on the incubation temperature, different types and quantities of plasma proteins were adsorbed on the NPs^11^. This means that plasma proteins interact with NPs to different extent. Change in protein corona decoration is partly related to the protein conformational changes occurred at high temperature^12^. Thus, it can be suggested that newly exposed epitopes/sequences, as a result of protein unfolding, determine how proteins interact with the EMT zeolite NPs.

**Table 2:** The Stern -Volmer constants calculated for fibrinogen–EMT zeolite NPs interactions.

Zeolite	T (K)	K_sv_ (M^−1^)	K_q_ (M^−1^ S^−1^)	R^2^
EMT NPs	298.15	6280.4	6280.4 × 10^8^	0.97
	310.15	4532.3	4532.3 × 10^8^	0.99
	313.15	3980.3	3980.3 × 10^8^	0.99
	315.15	3611.9	3611.9 × 10^8^	0.98

### Binding sites of protein on EMT zeolite nanoparticles

Fibrinogen showed different orientations on the NPs surface, which is depending on the physicochemical properties of NPs (surface charge, size, surface curvature), the type of presorted proteins and the degree of NP surface coverage^27,39,40^. For example, fibrinogen tends to be adsorbed on small-sized gold (5.6–14 nm) and silica (15–60 nm) NPs *via* side-on configuration^27,40^. However, fibrinogen preferably attached to NPs surface through end-on configuration as the size of NPs increased^40^. The number of binding sites per protein was calculated using double–logarithm equation (Fig. 2b and SI: Eq. S2)^41^. The proteins that adopt a side-on configuration wrap the NP surface and therefore, engage more binding sites compared to the end-on state. As expected, fibrinogen, which is a hydrophilic protein, has more than one, binding sites on EMT zeolite NPs (Table 3). It is suggested that fibrinogen is mainly adsorbed on EMT NPs through side-on configuration. The influence of incubation temperature on fibrinogen-NPs interactions is presented in Fig. 2b. The number of binding sites per protein decreased as the incubation temperature increased from 25 to 42 °C. Thus, the temperature-induced conformational changes affect the orientation of proteins on the NPs. The proteins adopted in the side-on/end-on configuration may reorient to end-on/side-on state after structural rearrangement. Depending on the protein unfolding extent and protein orientation model, the protein-NPs interaction may form different binding strengths. Therefore, various binding energies of the interaction at different temperatures can be explained by temperature-induced conformational changes.

**Table 3:** Binding parameters of fibrinogen–EMT zeolite NPs interactions at different temperatures.

Zeolite	T (K)	K_α_ (M^−1^)	*n*	R^2^
EMT NPs	298.15	1216746.20	1.56	0.98
	310.15	15595.52	1.13	0.99
	313.15	7184.55	1.06	0.99
	315.15	3322.76	0.99	0.98

### Cooperativity assay of fibrinogen and EMT zeolite nanoparticles

Binding kinetics and cooperativity in fibrinogen-zeolite NPs interaction were assessed using Hill equation (SI: Eq. S3). Hill coefficient determines whether different binding sites involved through protein-NP interaction are self-governed or cooperative. The calculated Hill coefficient in physiological like condition is near to 1 implying that single or multiple self-governing binding sites mediate the fibrinogen-zeolite interaction (Fig. 2c) (SI: Figs S1–S3)^42^. For non-cooperative protein binding, where Hill coefficient is near to 1, the affinity of proteins to NP surface is independent of the pre-sorbed proteins. Fibrinogen has multiple domains that potentially interact with different surfaces. For example, domains D and E illustrated different affinities to the same surface^43^. They also attached to zeolite NPs independently. It can be suggested that the adsorption and subsequent unfolding of each domain do not affect neighboring domain adsorption/structure.

The particle size plays crucial role in determining the cooperativity in NPs-protein interaction^40^. Considerable contradictories exist in the literatures regarding the effect of NPs size on the cooperativity in NPs-fibrinogen interaction. For example, Deng *et al*.^39^, showed positive cooperativity occurred when fibrinogen interacted with the gold NPs larger than 7 nm. In contrast, Lacerda *et al*.^44^, demonstrated that fibrinogen preferably bound to gold NPs larger than 5 nm in a negative cooperative manner.

### Analysis of thermodynamic parameters involved in the EMT zeolite nanoparticles and fibrinogen interactions

The thermodynamic parameters involved in fibrinogen–EMT NPs interaction were measured using fluorescent spectroscopy. Thermodynamic parameters such as enthalpy (ΔH), Gibbs free energy (ΔG) (Table 4), and entropy (ΔS) changes were indirectly calculated using the equations S4 and S5 (SI) to determine the governing forces on the fibrinogen−EMT NPs interaction. The negative values for ΔG indicate that the interaction occurs spontaneously. The negative values of ΔH (−2699.05 J mol^−1^ K^−1^) and ΔS (−787.16 J mol^−1^ K^−1^) imply that van der Waals (vdW) forces and hydrogen bonds are also involved in EMT−fibrinogen interaction^45^. Based on the exothermic reaction and the thermodynamic parameters obtained from van’t Hoff equation and corresponded plots of fibrinogen−EMT interactions, it is suggested that fibrinogen has a high affinity towards EMT NPs (Fig. 2d).

**Table 4:** Thermodynamic parameter involved in fibrinogen–EMT zeolite NPs interactions.

Zeolite	T (K)	ΔG (kJ mol^−1^)
EMT NPs	298.15	−34.72
	310.15	−24.88
	313.15	−23.11
	315.15	−21.22

### Secondary structure of bound fibrinogen on EMT zeolite nanoparticles

The secondary structure of fibrinogen was studied in the presence of various concentrations of EMT NPs using far-ultraviolet circular dichroism (CD) analytical approach. The CD spectra were recorded in the wavelength range 190−260 nm (SI: Figs S4–S7 and Tables S1–S10). Two negative peaks appeared at 208 nm and 222 nm; these peaks are characteristic of typical fibrinogen structure having α-helix (Fig. 2e). The reduced ellipticity at 208 nm and 222 nm indicates that the α-helix content of fibrinogen treated with NPs decreased significantly. This means that the secondary structure of fibrinogen significantly changes in the presence of EMT zeolite NPs (Fig. 2f). In addition, the degree of protein conformational changes gradually increased with increasing the NP concentration.

### Mechanistic understanding of fibrinogen-EMT zeolite interaction *via* molecular dynamics simulations

**Figure 3:**
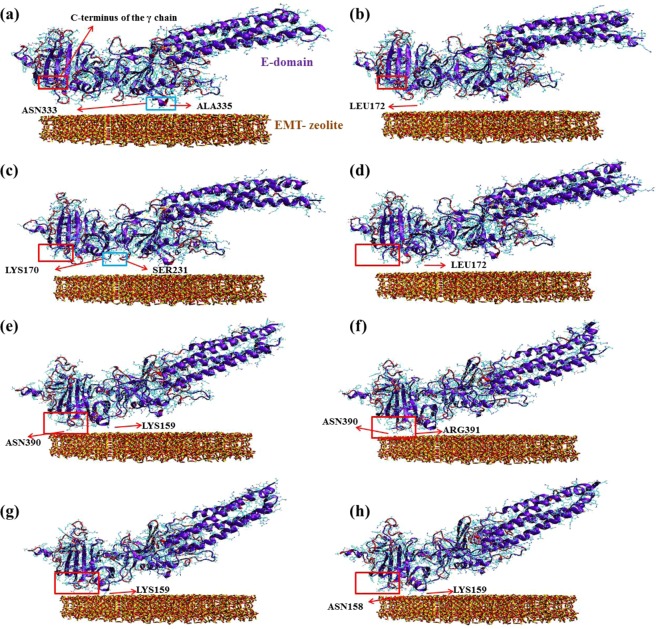
Interactions between D-domain and EMT zeolite NPs at different stages of simulation and residues involved in this interaction.

**Figure 4:**
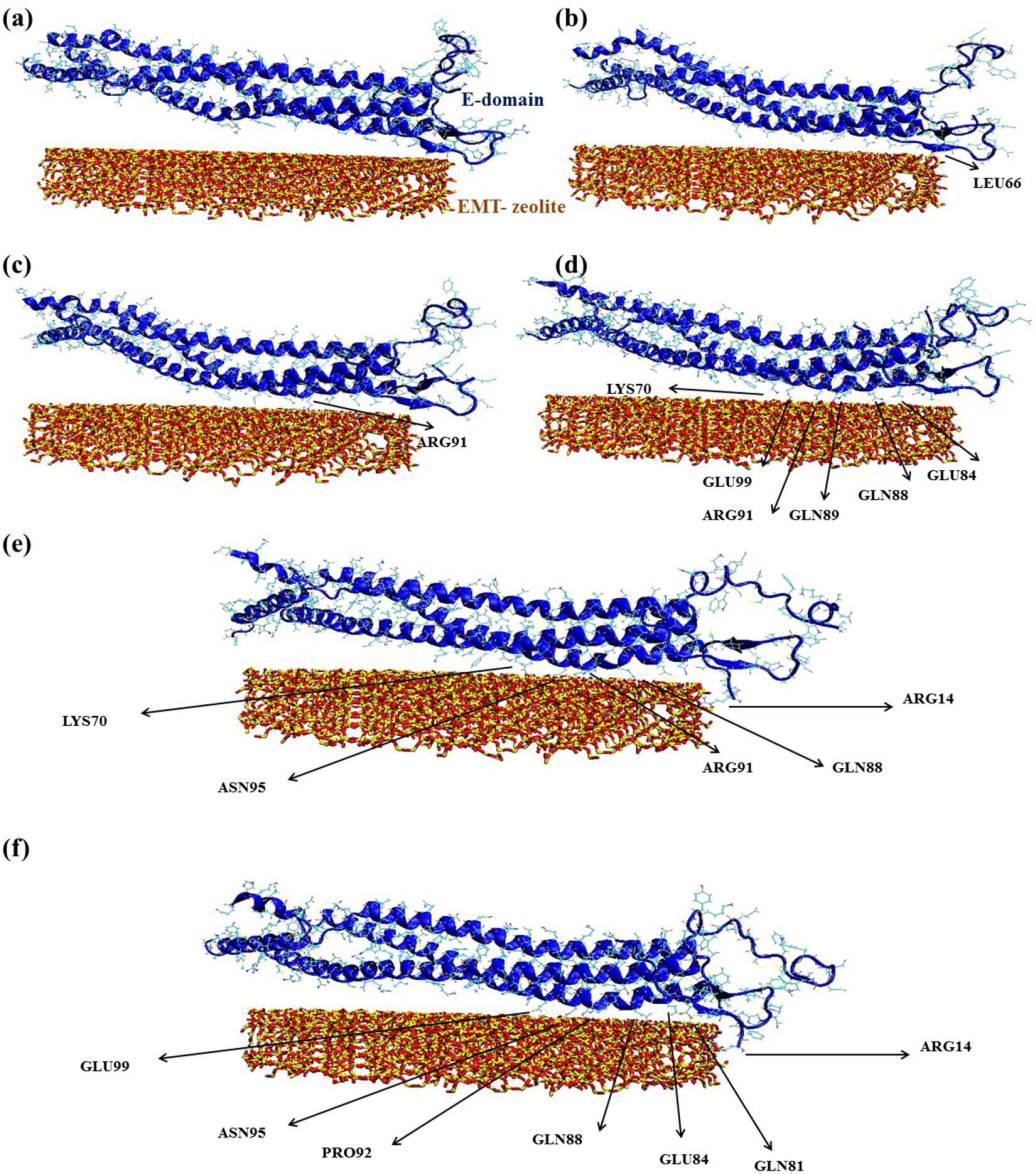
Interactions between E-domain and EMT zeolite NPs at different stages of simulation and residues involved in this interaction.

**Figure 5:**
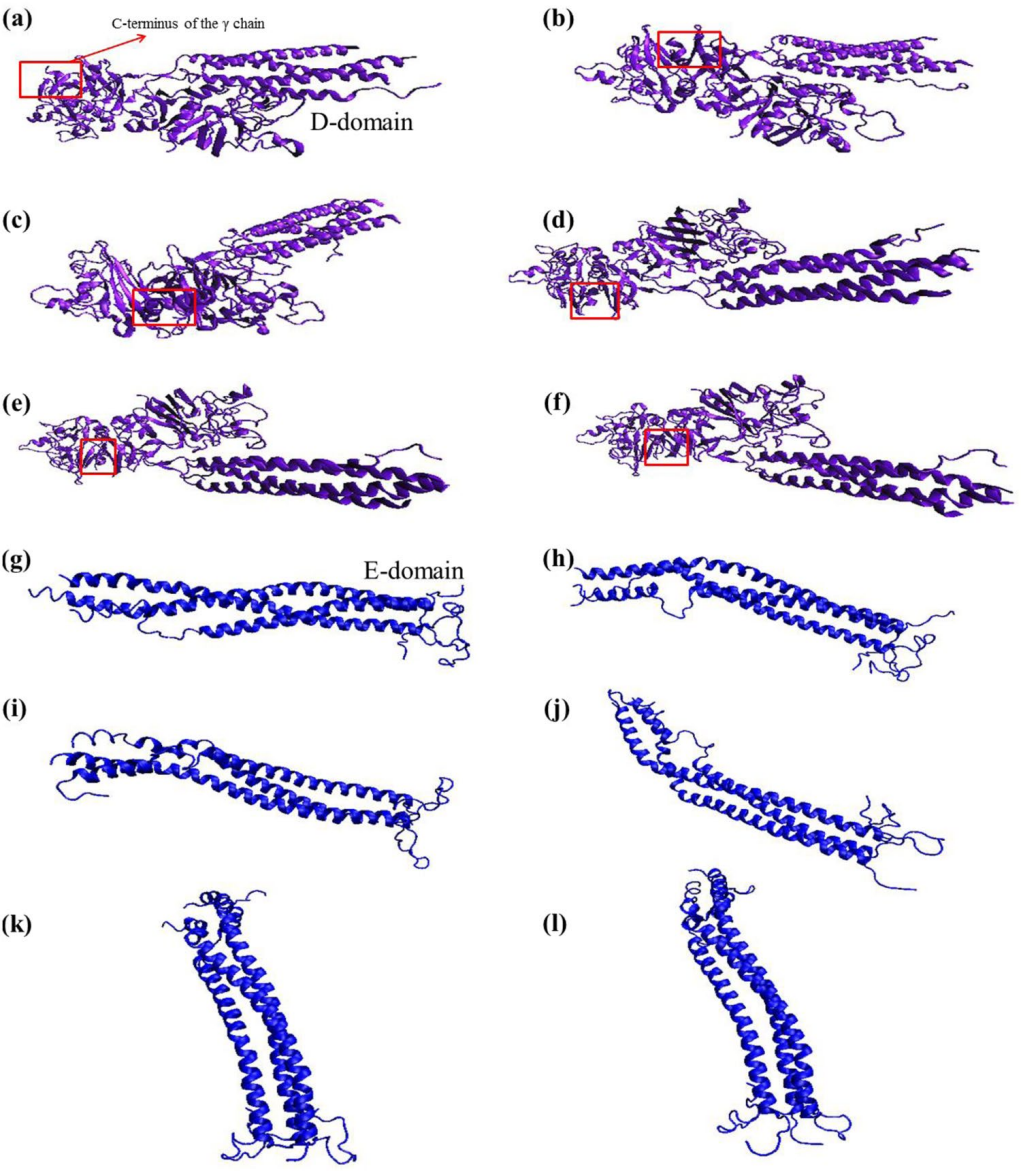
Snapshots of different steps of D-domain (**a**–**f**) and E-domain (**g**–**l**) simulations without EMT zeolite NPs as a control for calculating RMSD, RMSF and ∆SASA.

**Figure 6:**
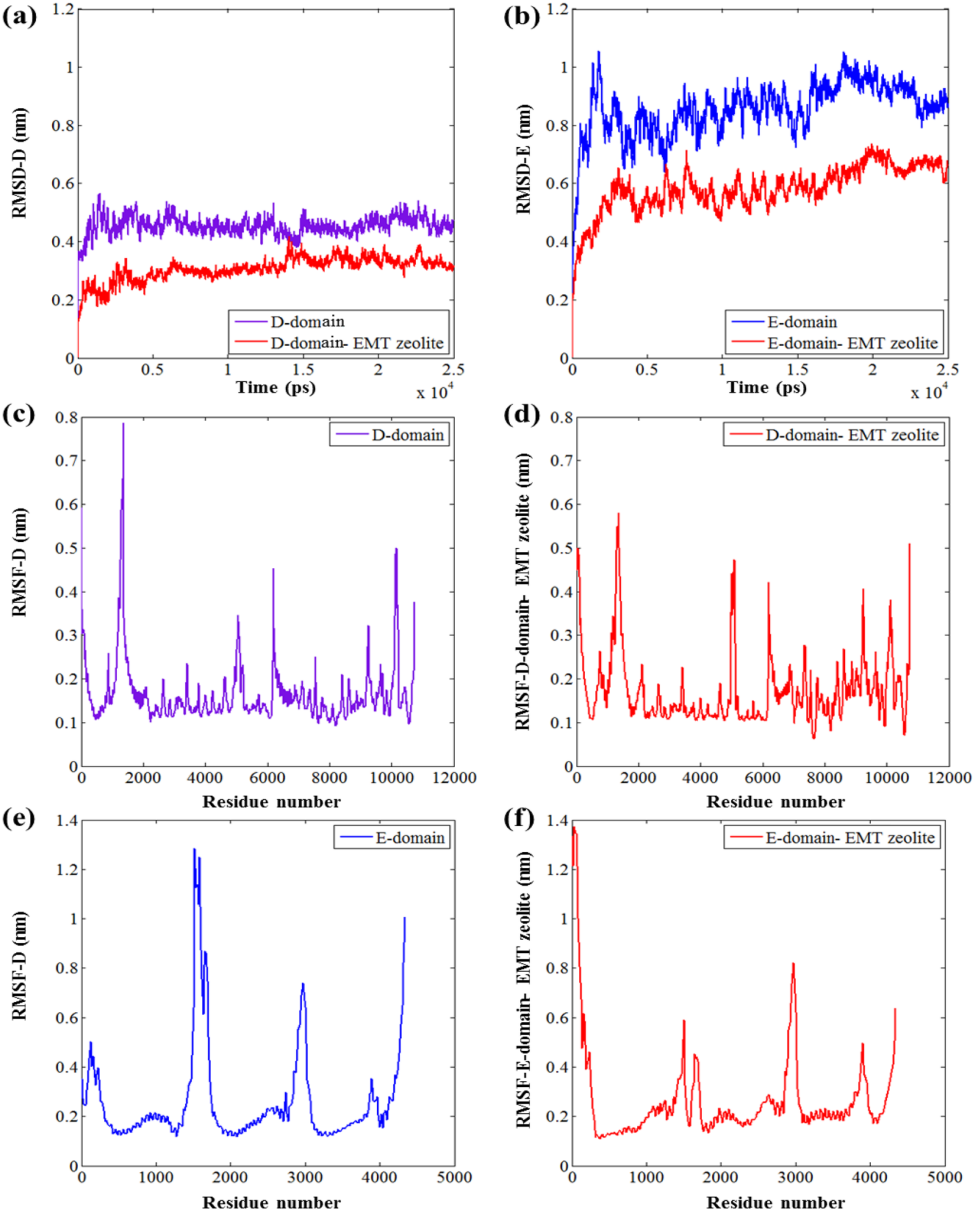
Root-mean-square deviation (RMSD) at equilibrium for (**a**) D-domain (**b**) and E-domain with/without EMT zeolite NPs. (**c**,**d**) Root-mean-square fluctuation (RMSF) per residue at equilibrium for D-domain-EMT zeolite and alone D-domain. These values are averaged over four simulations using each residue C_α_ for D-domain with/without zeolite. (**e,f**) RMSFs measured for the C_α_ of E-domain with/without zeolite.

**Figure 7:**
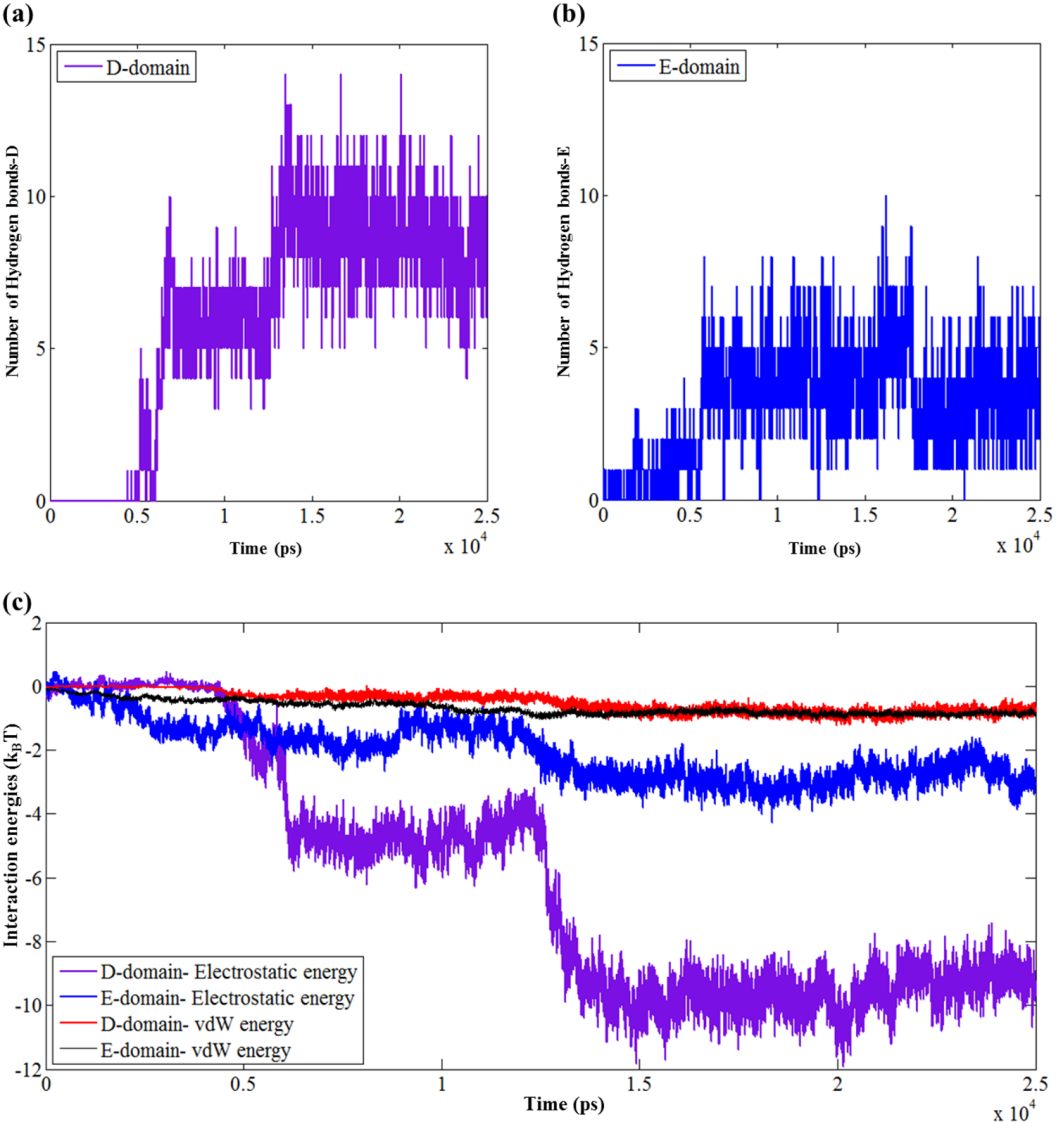
(**a**) Number of formed hydrogen bonds between D-domain and EMT zeolite NPs. (**b**) Number of formed hydrogen bonds between E-domain and EMT zeolite NPs. (**c**) Electrostatic and vdW energies between D-/E-domain and zeolite NPs. The important point is the difference between electrostatic energies of D-domain-EMT zeolite and E-domain-EMT zeolite which is about 4 k_B_T higher for D-domain.

The detailed mechanism of interactions between EMT zeolite NPs and fibrinogen D-/E-domains was studied using molecular dynamics (MD) simulations (Figs 3a–h and 4a–f). The MD simulation analyses showed that binding of fibrinogen to EMT zeolite can change the structure of fibrinogen. To investigate the pro-inflammatory potential of EMT zeolite in atomistic views, the simulation of fibrinogen D-domain was performed in the presence and absence (as a control) of zeolite NPs (Figs 3a–h and 5a–f). The root mean square deviation (RMSD) analysis showed that the D-domain has more stability in interaction with zeolite NPs compared to the D-domain alone. The root mean square fluctuations (RMSF) also decreased for D-domain interacted with the EMT zeolite NPs indicating the higher stability of the D-domain interacting with the EMT zeolite NPs (Fig. 6a,c,d). The most striking observation is that γ^377–394^ flexibility increased through interaction of D-domain with EMT zeolite NPs. The data obtained from this analysis revealed that at first, turns of β-chain (in D-domain) have critical role in binding of the D-domain to the EMT zeolite NPs. The non-polar (Ala335) and polar (Asn333) residues located in β-chain mediate the adsorption of protein onto the EMT zeolite surface (Fig. 3a). After binding of D-domain to EMT zeolite NPs, hydrophobic residue (Leu172) changes the configuration of D-domain possibly through hydrophobic interactions (according to the observed simulation outcomes; please see Fig. 3b for details) with other residues and EMT zeolite NPs. During simulations, other residues in γ-chain interact with EMT zeolite. Moreover, the interactions between turns of β and γ chains affect the orientation and exposure of residues. For example, the interaction of Ser231 located in β-chain with LYS170 located in γ-chain changes the orientation of D-domain on the surface of the EMT zeolite NPs (Fig. 3c). The Ser231-Lys170 interaction mediates binding of Leu172 to EMT zeolite surface *via* hydrophobic-hydrophobic interactions (Fig. 3d). Then, Asn390 forms hydrogen bonds with the surface and trigger the formation of hydrogen bonds between D-domain and EMT zeolite (Figs 3e and 7a). In addition, the positively charged Lys159 interacted with the negatively charged oxygen atoms in EMT zeolite through electrostatic interaction (Figs 3e,f,g and 7c). The γ-chain residues (*viz*. Lys159, Lys170, Lys173, Lys196, Asp199, Asp203, Lys206, Glu213, His217, Glu225, Glu231, Lys232, His234, Glu249, Glu251, Asp252, Asp26, Lys266, Glu270, Lys273, Asp285, Asp291, Asp294, Asp297, Asp298, Asp301, Lys302, His307, Asp320, Lys321, Glu323, Glu328, Asp330, Lys338, His340, His343, Lys356, Lys373, Lys380, Lys381) are involved in electrostatic interactions with EMT zeolite NPs (Fig. 7c).

**Figure 8:**
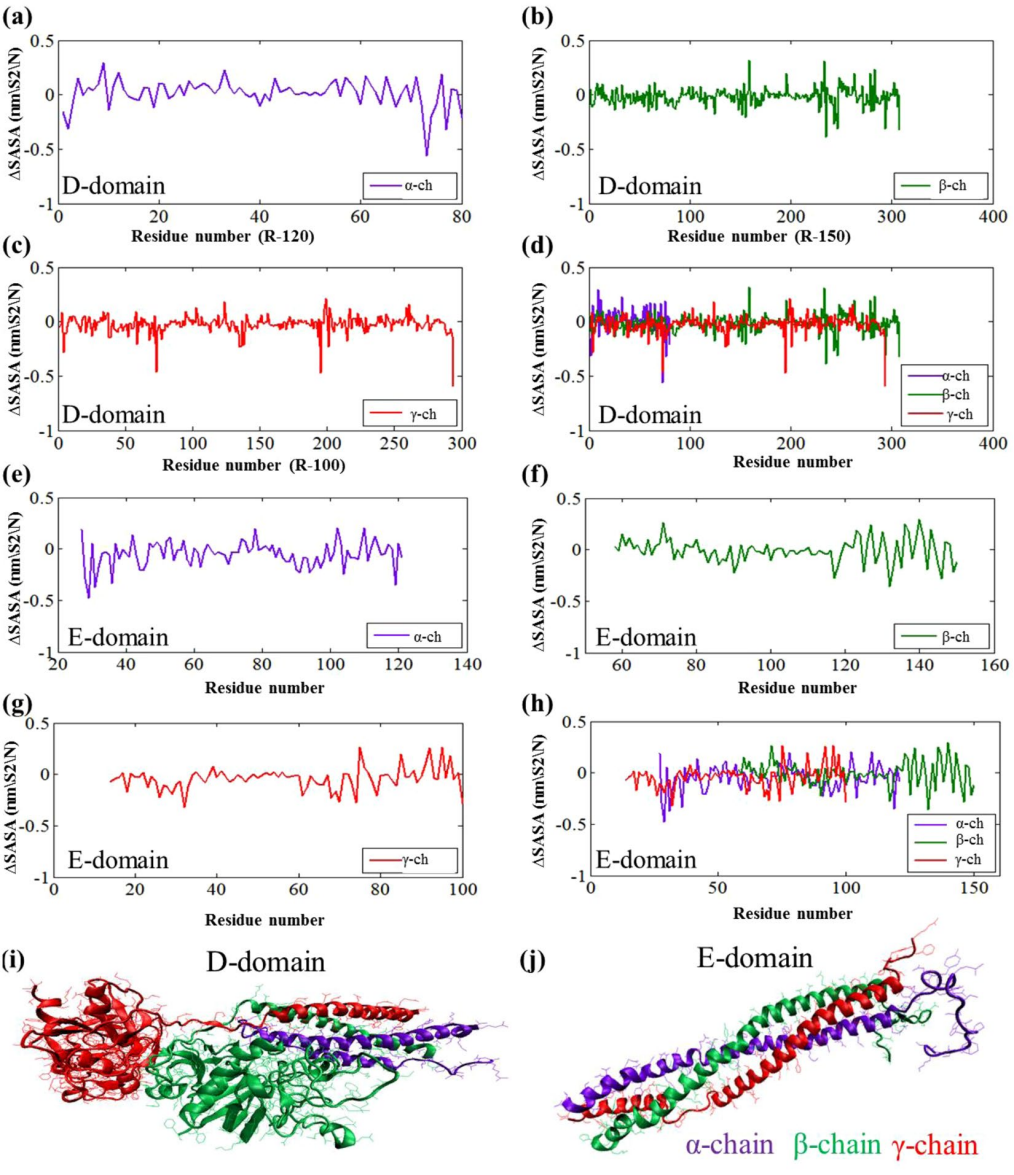
The differences of SASA for two setups (∆SASA = SASA^D-/E-domain^ − SASA^D-/E-domain- EMT zeolite^) with error bars of 0.01 are presented to identify important residues exposed when D and E-domains of fibrinogen interact with EMT zeolite (for α, β and the γ chains). (**a**) The ∆SASA for α-chain of D-domain. (**b**) The ∆SASA for β-chain of D-domain. (**c**) The ∆SASA for γ-chain of D-domain. (**d**) The ∆SASA for α/β/γ-chains of D-domain. (**e**) The ∆SASA for α-chain of E-domain. (**f**) The ∆SASA for β-chain of E-domain. (**g**) The ∆SASA for γ-chain of E-domain. (**h**) The ∆SASA for α/β/γ-chains of E-domain. (**i**) For D-domain of fibrinogen, α-chain is colored in violet, β-chain is colored in green and γ-chain is colored in red. (**j**) For E-domain of fibrinogen, α-chain is colored in violet, β-chain is colored in green and γ-chain is colored in red.

In addition to electrostatic interactions, stable hydrogen bonds are formed between polar residues (Asn158) and the EMT zeolite NPs (Fig. 3h). The consequence of all these events is exposing the C-terminus of γ-chain, γ^377–394^ (Tyr377-Ser378-Met379-Lys380-Lys381-Thr382-Thr383-Met384-LYS385-Ile386-Ile387-Pro388-Phe389-Asn390-Arg391-Leu392-Thr393 -Ile394) (Fig. 3). The solvent accessible surface area (SASA) was calculated to study the exposing of the C-terminus of γ-chain during interaction with zeolite NPs (Fig. 8a–d). The SASA was calculated for alone D-domain protein and D-domain interacted with the EMT zeolite NPs. The differences of SASA for two setups (∆SASA = SASA^D-domain^ − SASA^D-domain- EMT zeolite^) with error bars of 0.01 were presented to identify the important residues exposed when D-domain of fibrinogen interact with the EMT zeolite NPs (Fig. 8c,d). This analysis strongly confirms the contact of γ^377–394^ with the EMT zeolite NPs. As depicted in Fig. 8c,d, the γ^377–394^ showed the highest level of exposure (3σ) in comparison to other sequences/residues by considering the error bar (0.01). The exposure of residues from γ, β and α-chains are ranked in the following orders respectively: (γ) Ile394 > Phe295 > Lys173 > Asp104 > Asp301 > Trp315 > Lys159 > Leu235 > Ser237; (β) Trp385 > Phe458 > Lys396 > Trp444 > Gln393; (α) Leu193 > Arg197 > Leu122 > Gln200 (Fig. 8a–d).

To get deeper understanding on the interaction of fibrinogen with the EMT zeolite NPs, we also simulated E-domain of this protein (Figs 4a–f and 5g–l). The non-polar residue (Leu66) triggers the movement of E-domain towards the surface of the EMT zeolite NPs (Fig. 4a,b). In addition, the hydrophobic residues including Cys65, Phe74 and Ala68 are involved in this interaction *via* hydrophobic forces. It seems at early stages, the hydrophobic residues facilitate the interaction between E-domain and the EMT zeolite NPs. Indeed, binding of E-domain to the EMT zeolite is initiated and stabilized *via* hydrophobic and electrostatic forces, respectively (Fig. 7c). At initial steps, hydrophobic residues keep the E-domain near to zeolite and then polar (Gln88, Gln89 and Arg91) and charged (Lys70^α-chain^, Lys78^α-chain^, Lys81^β-chain^ Glu84^β-chain^ and Glu99^β-chain^) residues mediate the attachment of E-domain to the surface of the EMT zeolite (Fig. 4c,d). Arg14 and Arg91 also mediate the binding of E-domain to the EMT zeolite through interaction with NP surface, respectively (Fig. 4e). Moreover, Gln81, Glu84, Gln88, Asn95 and Glu99 residues interact with zeolite *via* electrostatic interactions and form hydrogen bonds (Fig. 4e,f). By calculating the vdW and electrostatic interactions, and also the hydrogen bonds between E-domain and zeolite, we found out the key role of negative charged residues (such as Glu84) in E-domain-zeolite interactions (Fig. 7). For D-domain, the positive charged residues, such as Lys159, keep fibrinogen in contact to zeolite for a long time, while for E-domain the negative charged residues, such as Glu84^β-chain^, play this role (Figs 3 and 4).

The RMSD and RMSF analyses showed that E-domain has more stable interaction with zeolite NPs compared to E-domain alone (Fig. 6b,e,f). Calculated electrostatic and vdW interactions between D/E-domain and EMT zeolite indicated that D-domain has more contribution in the interactions of fibrinogen with the EMT zeolite NPs than E-domain thanks to its high affinity to EMT zeolite exposed to the γ^377–394^ (Fig. 7c). Moreover, larger number of hydrogen bonds during the interaction of D-domain with the EMT zeolite NPs are formed compared to E-domain (Fig. 7a,b).

The SASA calculation was performed for E-domain in the presence and absence of the EMT zeolite in order to identify the residues exposed through interaction of E-domain with the EMT zeolite (Fig. 8e–h). In the case of E-domain, the exposure of residues from α, β and γ chains is ranked in following orders respectively: (α) Lys29 > Ser31 > Ile119 > Lys36 > Arg104 > Glu92 > Met91 > Lys44; (β) Val132 > Leu146 > Tyr117 > Glu90 > His149 > Trp125; (γ) Tyr32 > Ile100 > Ser74 > Pro70 > Tyr68 > Ala26 > Ile61.

## Discussion

The structural integrity of NPs bound proteins determines the *in vivo* fate of NP and biological responses. Understanding the NPs−protein interaction is crucial for predicting the therapeutic and/or toxic impacts of NPs *in vivo*. The structural changes in proteins, after association within the corona layer, can affect the cellular uptake mechanism of nanoparticles. For example, bovine serum albumin (BSA) proteins adsorbed on the surface of cationic polystyrene NPs experienced structural changes, while the bounds to anionic polystyrene remained intact^46^. These variations in the protein structure at the corona layer led to different interactions of the NPs with the cell receptors, i.e. the BSA-coated cationic and anionic NPs bounded to scavenger and native albumin receptors, respectively. In a similar study, Minchin group^47^ demonstrated that the silica NPs have capability to change the albumin’s structure, leading to exposure of a typical hidden epitope, which is exclusively recognized by macrophages expressing class A receptor. Prapainop *et al*.^48^, demonstrated that apolipoprotein conformation was changed after binding to quantum dots and it substantially increased their uptakes by macrophages. Thus, the challenges associated with the structural integrity of corona proteins raised serious concerns about the *in vivo* fate/behavior of NPs. Zeolite NPs have promising potential medical applications including drug delivery, imaging, and microbial infection and neurodegenerative diseases therapy^49–52^. Probing the structure of adsorbed proteins on the surface of zeolite NPs is of great interest as it helps scientists in the field to maximize the therapeutic efficacy of these particles while maintaining the toxic effects at a minimal level. This study revealed that the interaction between zeolite NPs and fibrinogen was strong, thermodynamically favorable and occurred in a non-cooperative manner. The EMT zeolite NPs considerably changed the secondary structure of fibrinogen.

MD simulations provide unique opportunity to understand the detailed mechanism of fibrinogen-EMT zeolite interactions. The data obtained from simulation studies had shown that fibrinogen binds to EMT zeolite through strong electrostatic and hydrophobic interactions. In fact, D- and E-domains, which have high affinity to EMT zeolite, are involved in this interaction. The binding affinity of D- and E-domains were calculated using linear interaction energy (LIE) method^53^. This analysis indicated that the affinity of D-domain to the surface of zeolite is 5 k_B_T higher than for the E-domain. This result implies that the D-domain has key role in binding of fibrinogen to EMT zeolite. D-domain plays the dominant role in binding of fibrinogen to EMT zeolite and undergoes highest level of structural changes leading to exposing the C-terminus of γ chain (γ^377–394^). Such exposure of the C-terminus of γ chain (γ^377–394^) is known to interact with integrin receptors which may in turn trigger inflammation response^24^. However, such inflammatory effects should be monitored in the actual *in vivo* conditions as the zeolite nanoparticles will interact with the entire plasma proteins and not the fibrinogen alone.

## Materials and Methods

### Synthesis and characterization of EMT zeolite NPs

The EMT zeolite NPs were synthesized as described previously^31^. Aluminate solution was prepared by mixing 9.074 g of sodium aluminate (Strem Chemicals), 1.61 g of sodium hydroxide (Prolabo, 97%) and 100 g of double distilled water. The mixture was stirred for 10 min before another 44.00 g of sodium hydroxide was added to the solution. The mixture was continuously stirred until a clear aluminate solution was obtained. The silicate solution was prepared by dissolving 57.692 g of sodium silicate (Prolabo, 27% SiO_2_, 8% Na_2_O) and 20.00 g of sodium hydroxide in 80.00 g of double distilled water. The preparation of both solutions are exothermic and hence both solutions should be cooled down in an ice bath (4 °C) prior mixing. The aluminate solution was then slowly poured into the silicate solution under magnetic stirring (800 rpm); a white colloidal suspension with a molar composition of 1Al_2_O_3_:5.15SiO_2_:18.45Na_2_O:240H_2_O was obtained. The resulting suspension was stirred for additional 5 min before it was crystallized at 30 °C for 36 h under static condition. The resulting EMT zeolite nanocrystals were then centrifuged (20000 rpm for 1 h) and purified with distilled water until the pH of the suspension reached 7.

The XRD patterns of zeolite NPs were obtained using a PANalytical X’Pert PRO XRD diffractometer (step size 0.01°, 1.5 seconds per step, Cu-K_α_ radiation). The morphology and crystallite size of zeolite samples were inspected by a transmission electron microscope (TEM) (JEOL Model 2010 FEG system, 200 kV). The average size of zeolite NPs was determined by randomly counting 50 particles through TEM observations obtained in different regions. The hydrodynamic size and zeta potential, ξ, of colloidal solution of zeolite NPs (1 wt%, pH = 7, 25 °C) were measured by a Malvern Zetasizer Nano Series equipment. The surface charge density, σ, was calculated using the Grahame equation (Eq. 1):

1 $${\rm{\sigma }}=\sqrt{8{{\rm{c}}}_{0}{{\rm{\varepsilon }}{\rm{\varepsilon }}}_{0}{{\rm{k}}}_{{\rm{B}}}{{\rm{N}}}_{{\rm{A}}}{\rm{T}}}\times \,\sinh (\frac{{{\rm{e}}{\rm{\Psi }}}_{0}}{2{{\rm{k}}}_{{\rm{B}}}{\rm{T}}})$$

where c_0_ is the concentration of zeolite (0.1%) in suspension in unit m^3^, εε_0_ is the dielectric permittivity of EMT zeolite (1.3547 × 10^−11^ AsV^−1^ m^−1^), k_B_ is the Boltzmann constant (1.381 × 10^−23^ J K^−1^), Ψ_0_ is the surface potential or zeta potential of the zeolite suspension (−45.7 mV), e is the electronic charge (1.602 × 10^−19^ C), and T is the absolute temperature (298 K).

The surface charge of the EMT NPs, Q, was calculated by Eq. 2:

2 $${\rm{Q}}=\frac{{\rm{\sigma }}\times {{\rm{S}}}_{{\rm{BET}}}}{{\rm{Si}}/{\rm{Al}}\,{\rm{ratio}}}$$

where S_BET_ is the specific surface area (m^2^ g^−1^) and Si/Al ratio is the silicon to aluminum ratio of the zeolite NPs.

The elemental composition of zeolite NPs was characterized by using a Phillips X’Unique X-ray Fluorescence (XRF) spectrometer. The porosity of zeolite NPs was analysed by a Micromeritics ASAP 2010 nitrogen adsorption analyzer. Prior to analysis, the powder was dehydrated at 250 °C under vacuum overnight. The specific surface area was calculated using the BET equation whiles the external surface area and micropore volume were computed using the *t*-plot technique. The pore sizes were calculated using the Density Functional Theory (DFT) model.

### Fluorescence spectroscopy

Fluorescence quenching of fibrinogen was measured in the presence of zeolite NPs with different concentrations using a Hitachi MPF-4 spectrofluorometer equipped with a thermostatically controlled cuvette compartment. The fluorescent property of fibrinogen is related to its aromatic amino acids such as tryptophan (Excitation: 280 nm; Emission: 360 nm). This analysis was performed at different temperatures (25, 37, 40 and 42 °C) to determine the effect of temperature on quenching process. The protein and NPs concentrations were 0.588 µM and 17–102 µM, respectively.

The fluorescence quenching was quantified by the following Eq. 3:

3 $${\rm{Q}}=({{\rm{F}}}_{0}-{\rm{F}})/{{\rm{F}}}_{0}$$

where F_0_ and F are fluorescence intensities in the absence and presence of NPs, respectively^44^. The association constant *K* is the reciprocal of the “dissociation constant”, k_D_. The Stern-Volmer equation (SI: Eq. S1) is used to assess the efficiency and mechanism of fluorescence quenching. By assuming that the NPs–protein binding occurs at the equilibrium condition, the quenching data was fitted for Q to determine an association constant (*K*_*α*_) to describe the NP–protein interaction (SI: Eq. S2). As fibrinogen potentially binds to the surface of zeolite NPs with different domains/binding sites, which displays the cooperativity in the binding equilibrium as expected. The binding kinetics and cooperativity in fibrinogen–zeolite interaction were determined using Hill equation (SI, Eq. S3).

### Circular dichroism (CD) spectroscopy

Fibrinogen (1 mg mL^−1^) was incubated with zeolite suspensions with different concentrations (concentration range from 4.25 to 119 µM) for 1 h at 37 °C. The CD spectra of resulting suspensions were recorded at the wavelengths between 190 and 260 nm with an average of 20 scans using a Aviv model 215 spectropolarimeter (Lakewood, NJ, USA). All CD measurements were performed at room temperature (25 °C), in a 1 mm path cuvette. The ellipticity was represented in milli degrees.

### Molecular dynamic simulation

All atomistic MD simulations were carried out using GROMACS 4.6.0 MD software and OPLSAA force field^54^. The OPLSAA force field was used for the EMT zeolite NPs, D-domain and E-domain with the TIP3P water model. The crystal structure of human fibrinogen was taken from Protein Data Bank under PDB entry 3GHG. The AutoDock Vina software was also used to perform the docking simulations^55^. These four setups (D-domain, D-domain-EMT zeolite, E-domain and E-domain-EMT zeolite) were minimized using steepest descent algorithm to remove any unfavorable interactions and then equilibrated in the NPT ensemble before 10-ns MD simulations in a neutralized aqueous solution (with the appropriate number of Na and Cl ions) in order to achieve biological conditions. The temperature and pressure were maintained in biological conditions (310 K, 1 bar) using V-rescale thermostat with a coupling time constant of 0.5 ps, and the Berendsen barostat with 0.5 ps coupling time constant after minimization steps. Time steps for all systems were 1 fs and neighbor lists were updated every 20 steps with a list cut off of 1.2 nm. In order to accurate coverage of long range interactions the particle mesh Ewald summation (PME) method with a direct space cut-off of 1.2 nm and a 4 cubic interpolation order was used^56^. In reciprocal space, 0.12 nm Fourier spacing (grid spacing for a fast Fourier transform) was used for controlling the wave vectors highest magnitude. For constraining bonds, the linear constraint solver (LINCS) algorithm was applied^57^. In order to integrate the motion equations, the leapfrog algorithm was used and simulations of all 4 setups (D-domain, D-domain-EMT zeolite, E-domain and E-domain-EMT zeolite) were replicated four times (all results were averaged). Visualizations of simulated systems including D-domain, D-domain-EMT zeolite, E-domain and E-domain-EMT zeolite were done using a VMD 1.9.3^58^.

## Supplementary information

Molecular interaction of fibrinogen with zeolite nanoparticles
