# Supplementary material for: Molecular interaction of fibrinogen with zeolite nanoparticles
Source: Sci Rep. 2019 Feb 7;9:1558. doi: 10.1038/s41598-018-37621-4 (PMC6367512; doi:10.1038/s41598-018-37621-4)
Supplement: Supplementary file 1 — Molecular interaction of fibrinogen with zeolite nanoparticles [file 41598_2018_37621_MOESM1_ESM.doc]

**(Supplementray Information)**

**Molecular interaction of fibrinogen with zeolite nanoparticles**

Hossein Derakhshankhah*^1, 2^*, Najla Hosseini*^3^*, Fereshteh Taghavi*^4^,* Samira Jafari*^2^*, Alireza Lotfabadi*^1,2,4^*, Mohammad Reza Ejtehadi*^5^*, Sahba Shahbazi*^6^*, Ali Fattahi *^2^*, Atiyeh Ghasemi*^4^*, Ebrahim Barzegari*^2^*, Mina Evini*^4^*, Ali Akbar Saboury*^4^*, Seyed Mehdi Kamali Shahri*^7^*, Behnaz Ghaemi*^8^*, Eng-Poh Ng*^9^*, Hussein Awala*^10^*, Fatemeh Omrani*^11^*, Iraj Nabipour*^11^*, Mohammad Raoufi*^12^*, Rassoul Dinarvand *^12^*, Koorosh shahpasand*^13^*, Svetlana Mintova*^10*^*, Mohammad Javad Hajipour*^11, 14*^* , and Morteza Mahmoudi *^15*^*

^1^ Nano Drug Delivery Research Center, Kermanshah University of Medical Sciences, Kermanshah, Iran.

^2^ Pharmacutical Sciences Research Center, Kermanshah University of Medical Sciences, Kermanshah, Iran.

^3^ Institute for Nanoscience and Nanotechnology and Center of Excellence in Complex Systems and Condensed Matter (CSCM), Sharif University of Technology, Tehran,1458889694, Iran.

^4^ Institute of Biochemistry and Biophysics, University of Tehran, Tehran, Iran.

^5^ Department of Physics, Sharif University of Technology, P. O. Box 11155-9161, Tehran, Iran and Center of Excellence in Complex Systems and Condensed Matter (CSCM), Sharif University of Technology, Tehran,1458889694, Iran.

^6^School of Biology College of Science, University of Tehran, Tehran, Iran

^7^ Department of Chemical Engineering, The Pennsylvania State University, University Park, PA 16802, United States.

^8^Department of Medical Nanotechnology, School of Advanced Technologies in Medicine (SATiM)

^9^School of Chemical Sciences, Universiti Sains Malaysia, 11800 USM, Malaysia.

^10^ Laboratory of Catalysis and Spectroscopy, ENSICAEN, University of Caen, CNRS, 6 Boulevard du Marechal Juin, 14050 Caen, France.

^11^ Persian Gulf Marine Biotechnology Research Center, the Persian Gulf Biomedical Sciences Research Institute, Bushehr University of Medical Sciences, Bushehr 75147, Iran.

^12^ Nanotechnology Research Center, Faculty of Pharmacy, Department of Pharmaceutical Nanotechnology, Faculty of Pharmacy, Tehran University of Medical Sciences, Tehran 13169-43551, Iran.

^13^Department of Brain and Cognitive Sciences, Cell Science Research Center, Royan Institute for Stem Cell Biology and Technology, ACECR, Tehran Iran

^14^ Non-Communicable Diseases Research Center, Endocrinology and Metabolism Population Sciences Institute, Tehran University of Medical Sciences, Tehran 13169-43551, Iran.

^15^ Department of Anesthesiology, Brigham and Women’s Hospital, Harvard Medical School, Boston, Massachusetts 02115, United States.

* Corresponding authors:

Email: (SM) [mintova@ensicaen.fr](mailto:mintova@ensicaen.fr); (MJH) [mj.hajipour@ncdrc.info](mailto:mj.hajipour@ncdrc.info); (MM) [mmahmoudi@bwh.harvard.edu](mailto:mmahmoudi@bwh.harvard.edu)

S1. Quenching effects of EMT NPs at different temperatures

In order to determine the molecular quenching mechanism, the fluorescence quenching results were analyzed by Stern-Volmer equation (Eq. S1).[^1^](#_ENREF_1)

F_0_/F = 1 + K_SV_ [Q] *= 1 + K_q_ τ* [Q] (Eq. S1)

F_0_ and F reveal the fluorescence intensities at the steady-state of fibrinogen in the absence and presence of quencher (EMT-type zeolite NPs), respectively. K_SV_ is the Stern-Volmer quenching constant and it is gained from the linear regression of Stern-Volmer equation; K_q_ is the quenching rate constant of protein whose maximum value is known to be 2.0 × 10^10^ L mol^-1^ s^-1^.[^2^](#_ENREF_2) [Q] is the concentration of quencher (EMT-type zeolite NPs). τ is the average lifetime of the fluorophore/biomacromolecule in the absence of the quencher.

The following double-logarithm equation (Eq. S2) provides more information about binding equilibrium.[^3^](#_ENREF_3)

Log ((F_0_−F)/F) = Log *K* + *n*Log [Q] (Eq. S2)

In this equation, *n* is the number of binding sites per protein and *K* is the association constant (K_α_).

S2. Cooperativity studies

The Hill coefficient was measured at different temperature using Hill equation (Eq. S3) through:

Ln ((F_0_-F)/F) = *n* Ln [Q] − *n* Ln k_D_ (Eq. S3)

where F_0_ and F reveal the fluorescence intensities of fibrinogen in the absence and presence of ligand (EMT-type Zeolite NPs), respectively. [Q] is the concentration of ligand (EMT-type zeolite NPs). *n* and k_D_ are Hill coefficient and the protein-NP binding constant in equilibrium, respectively. With regard to the Hill equation, in the plot of Ln ((F_0_-F)/F) versus Ln[Q], *n* is the slope of the curve. The obtained plots are shown below (Figs. S1-S3):


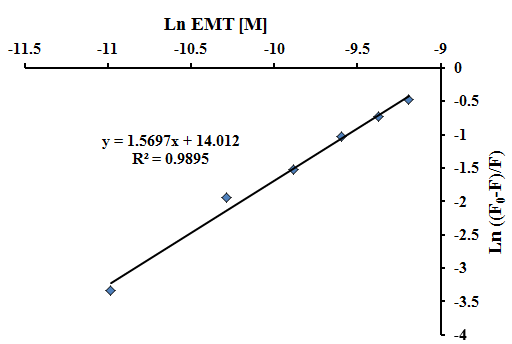


Fig. S1. Hill plot of the interaction between EMT zeolite NPs and fibrinogen at 25 °C.


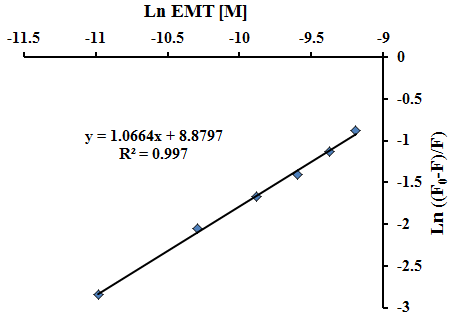


Fig. S2. Hill plot of the interaction between EMT zeolite NPs and fibrinogen at 40 °C.


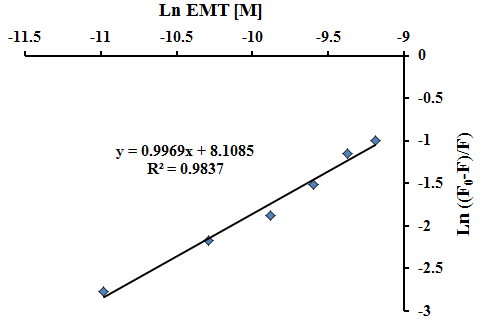


Fig. S3. Hill plot of the interaction between EMT zeolite NPs and fibrinogen 42 °C.

S3. Thermodynamic analysis

Free energy changes of the interaction between fibrinogen and NPs were calculated using following formula:[^4^](#_ENREF_4)

ΔG = –RTLn(K_α_) (Eq. S4)

In this equation, K_α_ is the association/binding constants for EMT NPs. T is the temperature of experiments and R is the gas constant (8.314 J mol^−1^ K^−1^). With assuming that ΔH and ΔS values do not vary significantly over the temperature, the ΔG was calculated from Gibbs equation (Eq. S4) and other parameters can be obtained by plotting the binding constant according to van’t Hoff equation (Eq. S5).[^5^](#_ENREF_5)

Ln (K_α_) = - (ΔH/RT) + (ΔS/R) (Eq. S5)

S4. Circular Dichroism data

Tables S1–S10 and Figures S4–S7 give the detailed information obtained from the CD spectroscopy on the intereaction between fibrinogen and EMT zeolite NPs.

Table S1. Percentage of different secondary structures in fibrinogen (0.25 mg mL^-1^).

|  | 195-260 nm | 200-260 nm | 205-260 nm | 210-260 nm |
| --- | --- | --- | --- | --- |
| Helix | 48.00% | 56.30% | 55.40% | 56.30% |
| Antiparallel | 3.50% | 2.10% | 2.20% | 2.30% |
| Parallel | 6.80% | 6.20% | 5.80% | 5.80% |
| Beta-Turn | 13.00% | 11.80% | 11.80% | 12.20% |
| Random Coil | 29.50% | 26.10% | 26.80% | 27.60% |
| Total Sum | 100.70% | 102.40% | 102.00% | 104.10% |

Table S2. Percentage of different secondary structures in fibrinogen treated with EMT zeolite NPs (25 μg mL^-1^).

|  | 195-260 nm | 200-260 nm | 205-260 nm | 210-260 nm |
| --- | --- | --- | --- | --- |
| Helix | 42.80% | 45.50% | 45.00% | 45.30% |
| Antiparallel | 5.60% | 4.40% | 4.50% | 4.60% |
| Parallel | 6.90% | 6.40% | 6.00% | 5.90% |
| Beta-Turn | 14.10% | 13.30% | 13.30% | 13.90% |
| Random Coil | 30.40% | 31.20% | 31.30% | 31.50% |
| Total Sum | 99.90% | 100.80% | 100.10% | 101.20% |

Table S3. Percentage of different secondary structures in fibrinogen treated with EMT zeolite NPs (50 μg mL^-1^).

|  | 195-260 nm | 200-260 nm | 205-260 nm | 210-260 nm |
| --- | --- | --- | --- | --- |
| Helix | 37.80% | 43.70% | 43.40% | 44.00% |
| Antiparallel | 7.90% | 4.80% | 4.90% | 5.00% |
| Parallel | 6.60% | 6.30% | 5.90% | 5.80% |
| Beta-Turn | 15.10% | 13.60% | 13.60% | 14.10% |
| Random Coil | 29.70% | 31.30% | 31.10% | 31.40% |
| Total Sum | 97.10% | 99.70% | 98.90% | 100.30% |

Table S4. Percentage of different secondary structures in fibrinogen treated with EMT zeolite NPs (100 μg mL^-1^).

|  | 195-260 nm | 200-260 nm | 205-260 nm | 210-260 nm |
| --- | --- | --- | --- | --- |
| Helix | 41.50% | 43.80% | 42.40% | 42.50% |
| Antiparallel | 7.00% | 5.30% | 5.60% | 5.80% |
| Parallel | 7.20% | 6.60% | 6.10% | 6.00% |
| Beta-Turn | 14.40% | 13.60% | 13.70% | 14.50% |
| Random Coil | 31.30% | 33.10% | 34.10% | 33.90% |
| Total Sum | 101.40% | 102.40% | 101.80% | 102.70% |

Table S5. Percentage of different secondary structures in fibrinogen treated with EMT zeolite NPs (200 μg mL^-1^).

|  | 195-260 nm | 200-260 nm | 205-260 nm | 210-260 nm |
| --- | --- | --- | --- | --- |
| Helix | 41.80% | 43.40% | 42.10% | 42.10% |
| Antiparallel | 7.50% | 5.50% | 5.70% | 6.00% |
| Parallel | 7.10% | 6.70% | 6.10% | 6.00% |
| Beta-Turn | 14.70% | 13.60% | 13.70% | 14.60% |
| Random Coil | 29.00% | 33.80% | 34.60% | 34.30% |
| Total Sum | 100.10% | 103.00% | 102.20% | 103.00% |

Table S6. Percentage of different secondary structures in fibrinogen treated with EMT zeolite NPs (300 μg mL^-1^).

|  | 195-260 nm | 200-260 nm | 205-260 nm | 210-260 nm |
| --- | --- | --- | --- | --- |
| Helix | 44.50% | 43.30% | 41.60% | 41.40% |
| Antiparallel | 6.70% | 5.60% | 6.00% | 6.40% |
| Parallel | 7.20% | 6.80% | 6.20% | 6.00% |
| Beta-Turn | 14.50% | 13.60% | 13.80% | 14.70% |
| Random Coil | 28.10% | 34.20% | 35.30% | 35.00% |
| Total Sum | 100.80% | 103.60% | 102.80% | 103.50% |

Table S7. Percentage of different secondary structures in fibrinogen treated with EMT zeolite NPs (400 μg mL^-1^).

|  | 195-260 nm | 200-260 nm | 205-260 nm | 210-260 nm |
| --- | --- | --- | --- | --- |
| Helix | 42.50% | 42.20% | 40.20% | 39.60% |
| Antiparallel | 8.00% | 6.30% | 6.70% | 7.20% |
| Parallel | 7.30% | 6.90% | 6.20% | 6.00% |
| Beta-Turn | 14.80% | 13.80% | 14.00% | 15.10% |
| Random Coil | 29.10% | 35.50% | 36.80% | 36.10% |
| Total Sum | 101.70% | 104.70% | 103.90% | 104.00% |

Table S8. Percentage of different secondary structures in fibrinogen treated with EMT zeolite NPs (500 μg mL^-1^).

|  | 195-260 nm | 200-260 nm | 205-260 nm | 210-260 nm |
| --- | --- | --- | --- | --- |
| Helix | 39.10% | 41.50% | 39.50% | 38.50% |
| Antiparallel | 10.60% | 6.90% | 7.20% | 8.00% |
| Parallel | 7.40% | 7.10% | 6.40% | 6.10% |
| Beta-Turn | 15.40% | 13.90% | 14.10% | 15.30% |
| Random Coil | 29.90% | 37.10% | 38.60% | 37.40% |
| Total Sum | 102.50% | 106.50% | 105.80% | 105.30% |
|  |  |  |  |  |

Table S9. Percentage of different secondary structures in fibrinogen treated with EMT zeolite NPs (600 μg mL^-1^).

|  | 195-260 nm | 200-260 nm | 205-260 nm | 210-260 nm |
| --- | --- | --- | --- | --- |
| Helix | 45.60% | 41.40% | 39.50% | 38.10% |
| Antiparallel | 7.20% | 7.10% | 7.40% | 8.30% |
| Parallel | 7.70% | 7.20% | 6.40% | 6.10% |
| Beta-Turn | 14.30% | 13.90% | 14.00% | 15.40% |
| Random Coil | 30.20% | 38.20% | 39.70% | 38.10% |
| Total Sum | 105.00% | 107.90% | 107.00% | 106.10% |

Table S10. Percentage of different secondary structures in fibrinogen treated with EMT zeolite NPs (700 μg mL^-1^).

|  | 195-260 nm | 200-260 nm | 205-260 nm | 210-260 nm |
| --- | --- | --- | --- | --- |
| Helix | 45.60% | 41.40% | 39.50% | 38.10% |
| Antiparallel | 7.20% | 7.10% | 7.40% | 8.30% |
| Parallel | 7.70% | 7.20% | 6.40% | 6.10% |
| Beta-Turn | 14.30% | 13.90% | 14.00% | 15.40% |
| Random Coil | 30.20% | 38.20% | 39.70% | 38.10% |
| Total Sum | 105.00% | 107.90% | 107.00% | 106.10% |

Alterations of different secondary structure types by increasing the EMT concentrations (μg mL^-1^), studied at various wavelength ranges, have been depicted in Figures S4−S7. Results are mean ± standard error of mean (*n* = 20). Asterisk means significant change compared to control (fibrinogen alone) at p < 0.05.


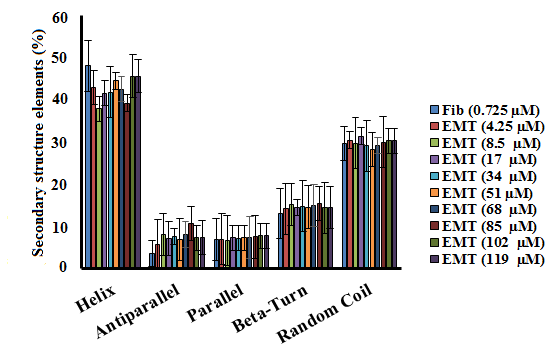


Fig. S4 Fibrinogen secondary structure content changes upon binding EMT zeolite NPs studied at 195−260 nm.


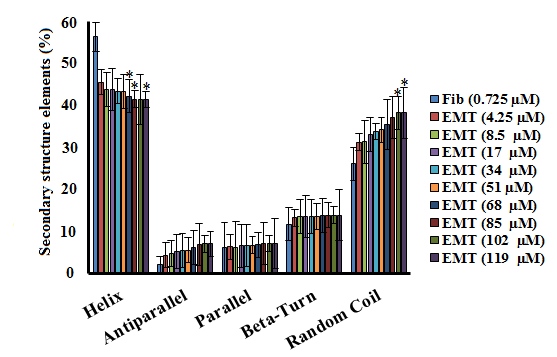


Fig. S5 Fibrinogen secondary structure content changes upon binding EMT zeolite NPs studied at 200−260 nm.


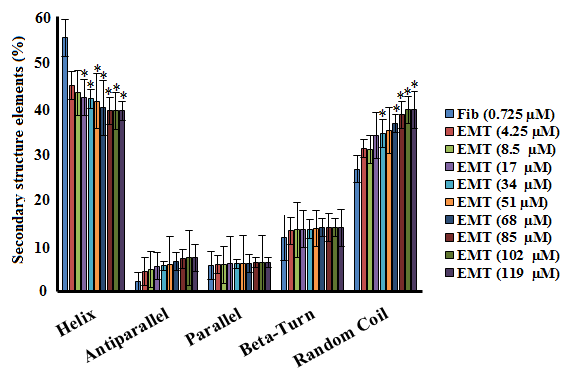


Fig. S6 Fibrinogen secondary structure content changes upon binding EMT zeolite NPs studied at 205−260 nm.


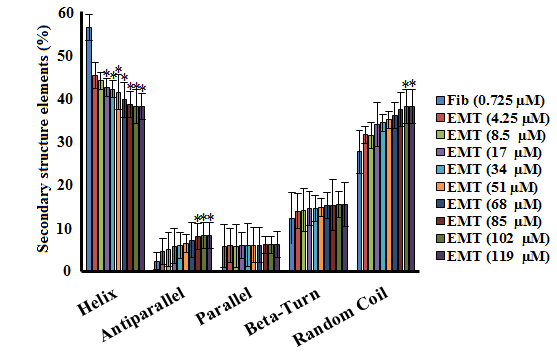


Fig. S7 Fibrinogen secondary structure content changes upon binding EMT zeolite NPs studied at 210−260 nm.

**Author contribution**

M. J. H. and M. M. proposed the original idea. H. D., F. T., S. J., A. R. L., S. S., A. F., A. G., E. B., M. E., A. A. S., S. M. K. S., B. G., F. O., I. N., M. R., R. D. and K. S. performed the experiments. N. H. and M. R. E. conducted the simulation analyses. E. P. N., H. A. and S. M. synthesized and characterized EMT zeolite. M. J. H designed the experimental and simulation analyses and wrote the manuscript. M. R. E., S. M. and M. M. assisted in interpreting analysis of the data, discussions and writing the manuscript.

**Conflict of Interest**

The authors have no conflict of interest to declare.

**Acknowledgement**

This work was supported by grants from the Kermanshah University of medical sciences and Bushehr University of medical sciences.

**References**

1 Zhou, Q. *et al.* Investigation on the interaction between a heterocyclic aminal derivative, SBDC, and human serum albumin. *Colloids Surf B.* **61**, 75-80 (2008).

2 Gerbanowski, A., Malabat, C., Rabiller, C. & Gueguen, J. Grafting of aliphatic and aromatic probes on rapeseed 2S and 12S proteins: influence on their structural and physicochemical properties.  *J. Agric. Food Chem.* **47**, 5218-5226 (1999).

3 Sklar, L. A., Hudson, B. S. & Simoni, R. D. Conjugated polyene fatty acids as fluorescent probes: binding to bovine serum albumin. *Biochem.***16**, 5100-5108 (1977).

4 Ross, P. D. & Subramanian, S. Thermodynamics of protein association reactions: forces contributing to stability. *Biochem.* **20**, 3096-3102 (1981).

5 Zhang, Z. & Tang, R. Synthesis and fluorescence properties of Tb (III) complex with a novel β-diketone ligand as well as spectroscopic studies on the interaction between Tb (III) complex and bovine serum albumin. *J. Mol. Struct.* **1010**, 116-122 (2012).
